# Fidelity is not easy! Challenges and guidelines for assessing fidelity in complex interventions

**DOI:** 10.1186/s13063-021-05322-5

**Published:** 2021-05-29

**Authors:** Liane R. Ginsburg, Matthias Hoben, Adam Easterbrook, Ruth A. Anderson, Carole A. Estabrooks, Peter G. Norton

**Affiliations:** 1 School of Health Policy & Management, Faculty of Health, York University, Toronto, Ontario M3J 1P3 Canada; 2 Faculty of Nursing, University of Alberta, Edmonton, Alberta T6G 1C9 Canada; 3 School of Nursing, University of North Carolina, Chapel Hill, North Carolina 27599-7460 USA; 4 Cumming School of Medicine, University of Calgary, Calgary, Alberta T2N 4N1 Canada

**Keywords:** Fidelity, Fidelity assessment, Fidelity methodology, Pragmatic group-level trials, Complex interventions

## Abstract

**Background:**

Fidelity in complex behavioural interventions is underexplored and few comprehensive or detailed fidelity studies report on specific procedures for monitoring fidelity. Using Bellg’s popular Treatment Fidelity model, this paper aims to increase understanding of how to practically and comprehensively assess fidelity in complex, group-level, interventions.

**Approach and lessons learned:**

Drawing on our experience using a mixed methods approach to assess fidelity in the INFORM study (Improving Nursing home care through Feedback On perfoRMance data—INFORM), we report on challenges and adaptations experienced with our fidelity assessment approach and lessons learned. Six fidelity assessment challenges were identified: (1) the need to develop succinct tools to measure fidelity given tools tend to be intervention specific, (2) determining which components of fidelity (delivery, receipt, enactment) to emphasize, (3) unit of analysis considerations in group-level interventions, (4) missing data problems, (5) how to respond to and treat fidelity ‘failures’ and ‘deviations’ and lack of an overall fidelity assessment scheme, and (6) ensuring fidelity assessment doesn’t threaten internal validity.

**Recommendations and conclusions:**

Six guidelines, primarily applicable to group-level studies of complex interventions, are described to help address conceptual, methodological, and practical challenges with fidelity assessment in pragmatic trials. The current study offers guidance to researchers regarding key practical, methodological, and conceptual challenges associated with assessing fidelity in pragmatic trials. Greater attention to fidelity assessment and publication of fidelity results through detailed studies such as this one is critical for improving the quality of fidelity studies and, ultimately, the utility of published trials.

**Trial registration:**

ClinicalTrials.gov NCT02695836. Registered on February 24, 2016

**Supplementary Information:**

The online version contains supplementary material available at 10.1186/s13063-021-05322-5.

Contributions to the literature

- Although comprehensive models of fidelity assessment exist, recent systematic reviews indicate fidelity frameworks are rarely used and both fidelity receipt and enactment are poorly assessed and reported. Our analysis suggests these gaps are due to numerous practical, methodological, and conceptual challenges with fidelity assessment.
- Through a comprehensive, theory-based examination of fidelity in a complex, behavioural intervention (the INFORM trial), this study identifies six practical, methodological, and conceptual challenges with fidelity assessment and suggests six guidelines for improving the quality of fidelity studies.
- Study findings underscore the need for greater attention to fidelity and the need for more robust approaches to fidelity assessment, particularly with complex group-level interventions where there tends to be multiple components, smaller samples, and unit of analysis intricacies compared to simple interventions.

## Background

Fidelity is the extent to which an intervention is implemented as intended [1], and it provides important information regarding why an intervention may or may not have achieved its intended outcome. Accurate assessment of fidelity is crucial for drawing unequivocal conclusions about the effectiveness of interventions [2] (internal validity) and for facilitating replication and generalizability (external validity) [3, 4]. Ignoring fidelity increases the risk of discarding potentially effective interventions that failed to work because they were poorly implemented or accepting ineffective interventions where desired effects were achieved but were caused by factors other than the intervention. Fidelity is particularly important and challenging in complex interventions where there are multiple interacting components compared to less complex interventions [2] and where multiple actors may further complicate measurement of fidelity (e.g. in the event that different stakeholder or rater assessments of fidelity differ) [5]. Indeed, the UK Medical Research Council’s (MRC) recent guidance on process evaluations in complex trials is clear that an understanding of fidelity is needed to enable conclusions about intervention effectiveness [6].

Two dominant models that are useful for comprehensively assessing fidelity include the framework for evaluation of implementation fidelity proposed by Carroll et al. [7], and the treatment fidelity model proposed initially by Lichstein and colleagues [8], and later by Bellg and colleagues [3]. Others in the field of education [9] have proposed a model similar to the treatment fidelity model. One important difference between these models is that the framework proposed by Carroll et al. [7] treats ‘participant responsiveness’ as a *moderator* of fidelity while others treat it as a *component* of fidelity. Importantly, several components of fidelity are common to all of these models and focus on the *delivery* of an intervention (i.e. delivering an intervention consistently and as per the protocol to target persons who are to implement or apply behaviours of interest) as well as *receipt* (comprehension by target persons) and *enactment* of intervention activities (implementation of various target behaviours).

Although fidelity has gained increased attention in the literature over the last 30 years—and particularly since publication of the MRC guidance on process evaluations in complex trials [6]—its assessment is complex and onerous [2] and there are few comprehensive and detailed fidelity studies in the literature that evaluate delivery, receipt, and enactment (for some exceptions, see Keith et al. [10], Sprange et al. [11], and Carpenter et al. [12]). Studies of implementation fidelity have focused more on ‘delivery’ than ‘enactment’ [2, 3, 13, 14]. According to a recent systematic review of the quality of fidelity measures, only 20% of studies employed a fidelity framework and fewer than 50% of studies measured both fidelity *delivery* and fidelity engagement (which includes *receipt* and *enactment*) [2]. High-level approaches [9] and a compendium of methods suitable for assessing different aspects of fidelity (e.g. delivery, receipt, enactment) including checklists, observation, document analysis, interviews, and other approaches have been suggested in the literature [3], as have some recent guidelines for fidelity [5, 14]. For instance, Toomey and colleagues [14] make seven recommendations including the need to report on and differentiate between fidelity enhancement strategies and fidelity assessment strategies. Walton and colleagues outline a systematic approach for developing psychometrically sound approaches to broadly assess fidelity, including receipt and enactment, in the context of a complex intervention to promote independence in dementia [5]. Overall, however, most fidelity studies do not outline or report on *specific* procedures for monitoring fidelity [14–16].

Finally, many health services interventions seek to change practices at the group/unit-level. However, most fidelity studies are for interventions that target individual-level behaviour change (for exceptions see the fidelity study of a group-based parenting intervention by Breitenstein et al. [17]). Sample size challenges and aggregation questions may arise when the unit of analysis is the group. Analysis challenges can also arise when analysing multi-level fidelity data [18, 19] (e.g. when *program-level* fidelity delivery data and *individual participant-level* data on fidelity receipt or enactment need to be incorporated into fidelity analyses). Butel and colleagues [20] provide a useful example of a multi-level fidelity study of a childhood obesity intervention; however, multi-level fidelity challenges are generally not well addressed in the literature and further signal the need to attend to unit of analysis issues in fidelity assessment.

These literature gaps suggest fidelity research is needed in at least three areas: (1) understanding participants’ enactment of intervention activities, particularly for pragmatic trials where enactment is complex and is influenced by numerous contextual and other factors; (2) the practical application of comprehensive fidelity assessment models, and challenges assessing fidelity in pragmatic trials in particular; and (3) assessing fidelity in group-level interventions.

The current paper aims to contribute knowledge regarding how to practically and comprehensively assess fidelity in complex group-level interventions by drawing on our experience using a mixed methods approach to assess fidelity in the INFORM study (Improving Nursing home care through Feedback On perfoRMance data)—a large cluster-randomized trial designed to increase care aide involvement in formal team communications about resident care in nursing home settings [21]. Empirical results of the INFORM fidelity and effectiveness studies are reported elsewhere [22, 23]. In the current paper, we reflect on our approach to fidelity assessment in INFORM and describe fidelity challenges, adaptations, and lessons learned. We also suggest a set of guidelines (applicable primarily to group-level studies of complex interventions) to help address practical, methodological, and conceptual challenges with fidelity assessment in pragmatic trials.

### The INFORM study

Care aides (also known as health care aides) provide personal assistance and support to nursing home residents. In Canada, care aides are an unregulated group [24] who make up 60–80% of the nursing home workforce and provide a similar proportion of care in these settings [25, 26]. Their involvement in formal care communications is a key marker of a favourable work context which has been shown to be positively associated with best practice use [27] and resident outcomes [28]. Care aide involvement in formal care communications is also related to job satisfaction and reduced turnover [29]. However, within the nursing home sector evidence suggests that relationships and communication between care aides and regulated care providers (primarily nurses) are sub-optimal [30–34] and that care aides are rarely involved in decisions about resident care [35].

The INFORM intervention targets front-line managers and has two core components [21] which are based on goal setting [36] and social interaction theories: (1) goal setting activities designed to help managers and their team to: set specific attainable performance goals to improve care aide involvement in formal team communications about resident care, specify strategies for goal attainment and measure goal progress (the feedback element in goal setting theory) and (2) opportunities for participating units to interact throughout the intervention to share progress and challenges and learn effective strategies from one another.

In early 2016, baseline data on care aide involvement in formal team communications about resident care and other measures of context were collected and fed back, using oral presentations and a written report, to 201 care unit teams in 67 Western Canadian nursing homes. Homes were subsequently randomized to one of three INFORM study arms: *simple feedback* (control) care homes received only the oral and written report already delivered. One hundred six nursing home care units (clustered in 33 different nursing homes; range of 1–10 units per home, median = 3) assigned to *basic assisted feedback* and *enhanced assisted feedback* arms were invited to attend three workshops over a 1-year period. Workshops included a variety of activities to help with goal setting and goal attainment, such as support from facilitators, progress reporting by participating units, and inter-unit networking opportunities. In the enhanced assisted feedback arm, all three workshops were face to face. In the basic assisted feedback arm, the first workshop was face to face and the second and third were conducted virtually using webinar technology thus varying the extent of social interaction with peer units between these two study arms. Virtual workshops were 1.5 h—half the length of the face-to-face workshops.

Main trial results showed a statistically significant increase in care aides’ involvement in formal team communications about resident care in both the basic and enhanced assisted feedback arms compared to the simple feedback arm (no differences were observed between the basic and enhanced assisted feedback arms) [22]. Fidelity of the INFORM intervention was moderate to high, with fidelity delivery and receipt higher than fidelity enactment for both study arms. Higher enactment teams experienced a significantly larger improvement in formal team communications between baseline and follow-up compared to lower enactment teams [23].

### Fidelity in INFORM

Fidelity was assessed in the 106 nursing home care units randomized to the basic and enhanced assisted feedback arms. Guided by the treatment fidelity model outlined by Bellg and colleagues [3], a variety of quantitative and qualitative data on INFORM deliverers’ and participants’ activities and experiences were captured throughout the trial to assess fidelity. These data, gathered using checklists, debriefings, observation, exit surveys, and post-intervention focus groups (conducted 1 month following the final workshop), were used to measure fidelity delivery, receipt, and enactment as outlined in Table 1. Interested readers can find our fidelity data collection tools in supplemental appendices 1–6. Details regarding quantitative measures of fidelity referred to in Table 1 are described elsewhere [23].

**Table 1:** Treatment fidelity components and assessment approaches

Treatment fidelity components [3]	Approach to fidelity assessment	Supplemental appendix
Fidelity of treatment design		
• *Intervention is defined and operationalized consistent with its underlying theory*	• Intervention approach and components designed with careful consideration of underlying theory	n/a
Fidelity delivery		
• *Adherence to protocol (components, timing, etc.)*	• Delivery checklist completed by the same administrative staff person in each workshop to verify components were delivered as per protocol	1
• *Consistent delivery within study arm*	• Debriefings of workshop teams after each workshop regarding any deviations from protocol	1
• *Minimize contamination*	• Study designed to minimize contamination between study arms by (a) delivering separate workshops to participants in the basic and enhanced study arms and (b) asking participants not to talk about study details to persons external to their facilities	n/a
Fidelity receipt		
• *Participants understood/can use intervention components*	• Workshop attendance lists used to assess unit representation in each workshop (receipt only)	n/a
	• Coding of goal setting worksheets completed at 1st workshop for evidence of comprehension of goal setting and strategy development (receipt only)	2
Fidelity enactment • *Engagement in INFORM activities (adherence)*		
	• Exit survey data from workshop participants • Structured^†^ observation of workshop 2 team progress presentations (showing work to date)	3
	• Analysis of slide decks teams were asked to prepare showing progress to date	4
	• Mid-^‡^ and post-intervention focus groups	5
	• Expert assessment (enactment only)	6

^†^Added part way through the intervention when mid-intervention focus groups were cancelled; _‡_ planned but not carried out. These changes are discussed in the results section

## Approach and lessons learned

Because fidelity measures tend to be intervention-specific, this paper focuses on broader learnings regarding fidelity assessment. Lessons learned and guidelines proposed in this paper emerged from reflections and discussions regarding fidelity assessment, and subsequent adaptations, that were made by the authors on an ongoing basis throughout the INFORM trial. In particular, lessons learned and guidelines proposed are the result of (1) observations and reflections captured in field notes taken by authors of this paper (all of who were involved in delivering INFORM or evaluating fidelity); (2) fidelity discussions that took place among the INFORM researchers prior to and following each of the INFORM workshops; and (3) research team discussions that took place following the INFORM study as the authors began to think about fidelity assessment in the context of a different complex, group-level intervention in nursing homes (ClinicalTrials.gov NCT03426072).

### Challenges and lessons learned

Several aspects of the fidelity assessment approach described in Table 1 proceeded as planned (e.g. exit surveys were completed and returned by all participants at the initial workshop, a detailed delivery checklist was completed by workshop facilitators at the close of each workshop). However, we found that just as complex interventions are rarely implemented as per protocol, fidelity assessment in complex interventions often deviates from fidelity assessment protocol. We describe six fidelity assessment challenges that arose and how we continued to assess fidelity given eventualities inherent in a pragmatic trial. These challenges were experienced at one or more of the following stages of the trial [14]: (1) trial planning—identification/development of approaches and tools to assess fidelity, (2) trial conduct—application of fidelity assessment approaches, and (3) post-trial—during analysis and interpretation of fidelity data (see Table 2).

**Table 2:** Fidelity assessment challenges at different stages

	Stage of fidelity assessment challenge:		
	*Trial planning*	*Trial conduct*	*Post-trial*
*Fidelity assessment challenge*	*(1) Identification/development of approaches/tools*	*(2) Application of the fidelity approaches*	*(3) Analysis and interpretation of fidelity data*
1. Developing succinct tools to measure fidelity	✓✓✓	✓✓	✓
2. Deciding which components of fidelity to emphasize	✓✓✓	✓✓	
3. Unit of analysis considerations in group-level interventions	✓✓✓	✓✓	✓
4. The missing data problem		✓✓✓	✓✓✓
5. Responding to, and treating, fidelity ‘failures’ and ‘deviations ‘and a lack of an overall fidelity assessment scheme			✓✓✓
6. Ensuring that fidelity assessment does not threaten internal validity	✓✓✓	✓	

✓✓✓ challenge felt most strongly, ✓✓ challenge felt somewhat, ✓ challenge felt least strongly

### Fidelity assessment challenge 1: developing succinct tools to measure fidelity

Our first challenge was to determine how to operationalize/measure each component of fidelity and what specific tools to use. Because fidelity measures tend to be intervention-specific and generic validated measures of fidelity are generally not found in the literature, it quickly became apparent that we would need to develop tools from scratch. Developing succinct fidelity tools that reflect the constructs of interest was time consuming and some approaches required revision part way through the trial. Exit surveys designed to measure enactment of pre-workshop activities and receipt of workshop one concepts had to be analysed and refined prior to the second workshop. Observation tools designed to measure fidelity receipt and enactment at the second workshop had to be tested with observers (study team members) and observers had to be trained on use of the tool to ensure all had a common understanding of the response categories. Coding of Goal setting worksheets designed to measure receipt of workshop material required two of the researchers to develop a coding scheme, agree on boundaries for what constitutes ‘low’ and ‘high’ receipt, and resolve initial coding disagreements. Cohen’s Kappa, run to determine agreement on an initial 20 data elements from 5 goal setting worksheets, was slight (e.g. < 0.2). Following open discussion to gain a collective understanding of the codebook, Kappa was excellent with 100% agreement between the raters for the next 20 data elements coded. Overall, however, to achieve rigour in fidelity assessment the ongoing work to analyse and revise fidelity assessment approaches throughout the trial was demanding. We managed this largely by including several research team members who have expertise in survey design and measurement. High levels of researcher engagement throughout the trial were critical for success.

### Fidelity assessment challenge 2: deciding which components of fidelity to emphasize

Following the initial workshop, we felt we focused too heavily on assessment of fidelity delivery and not enough on fidelity receipt and enactment. The fidelity delivery checklist used in the first workshop covered delivery in a highly detailed way and was completed by several researchers present at the workshop; however, because nearly all items on the delivery checklist were under the control of one researcher and one trained facilitator who facilitated all of the workshops, fidelity of delivery was high—with little variation across raters and study regions. Four raters who rated each of 13 delivery fidelity checklist items largely agreed that none of the 13 criteria was violated. Conditional probabilities of all 4 raters agreeing that the respective criterion was met ranged between 75% (1 item) and 97% (5 items). Delivery checklists for workshops two and three were therefore shortened to contain only the most critical elements. Immediately following each workshop, a qualitative debrief discussion took place among study personnel present at the workshop to identify any deviations from delivery of the core intervention components. None were noted.

During workshops two and three, greater emphasis was placed on assessment of fidelity receipt and enactment. During the second workshop, receipt and enactment were assessed by members of the research team using structured observations of the progress presentations each unit was asked to deliver. At the close of workshop three, the two workshop facilitators used a consensus approach to arrive at a global enactment rating reflecting the extent to which each unit team enacted strategies to increase care aide involvement in formal care communications. Global rating scales have been shown to provide a faithful reflection of competency when completed by subject-matter experts in the context of Objective Structured Clinical Exams [37, 38]. This approach was adapted and used to obtain a single comprehensive measure of enactment given limitations associated with other approaches (enactment ratings based on observations are limited to what is included in the observed presentation and are costly; enactment ratings based on interviews are onerous and are subject to social desirability bias). The global enactment rating was a positive predictor of improvement in INFORM’s primary outcome [23].

### Fidelity assessment challenge 3: unit of analysis considerations in group-level interventions

Involving care aides in formal care communications required strategies and changes at the level of the care unit. The target of the intervention, as well as the unit of analysis in the INFORM cluster randomized trial, was at the group level (the resident care team on a care unit). Managers on participating care units were encouraged to attend the INFORM workshops and to bring 1–3 other unit members who they deemed appropriate for working on increasing care aide involvement in formal care communications. Some units brought educational specialists or Directors of Care who work across units in a facility; others brought care aides and/or nurses. Three fidelity assessment challenges arose with the unit of analysis being at the group level.

First, units often sent different staff members to the three intervention workshops and, in some cases, they sent only one person. In other instances, one individual represented more than one unit in a facility (e.g. a manager who had administrative responsibility for two or more units). If different unit team members show up to different parts of the intervention, should this component of fidelity enactment be rated as low? How should workshop attendance rates be calculated? How does consistent participation of the same persons versus inconsistent participation affect enactment of the intervention in the facilities, and how is intervention success affected? In a pragmatic trial where the unit of analysis is at the group level, we realized that nursing homes’ INFORM team membership was fluid. Accordingly, the workshop attendance component of fidelity enactment was deemed to have been met provided a unit had representation at each of the three workshops.

Second, for workshops two and three we asked each unit *team* to complete a single exit survey; however, exit survey data from the first workshop were collected at the individual level (we later aggregated workshop 1 exit survey data to the unit level for analysis). Workshop evaluations could be considered individual or group-level constructs and it is therefore important to be cognizant of unit of analysis issues in all fidelity data collection decisions.

Third, for feasibility reasons, not all fidelity assessment methods were carried out at the team level. In our post-intervention focus groups, data on fidelity receipt and enactment were gathered and examined by study arm because it was not feasible to conduct a focus group with each participating team for two reasons: (a) there were close to 100 teams and it is likely only 1–2 team members would turn out for a focus group, and (b) we did not want to include managers and staff in the same focus group since groups are ideally made up of homogeneous strangers [39] to avoid power differences within a single focus group. Instead, several separate manager and staff focus groups were conducted within each study arm, and it was not possible to identify which participants were speaking when during these focus groups. To balance unit of analysis considerations with data collection realities, post-intervention focus group fidelity data could be utilized to *broadly* understand the enactment process, rather than to assign fidelity enactment scores to each unit team. With additional resources and a slightly different process, data from individuals participating in mixed team focus groups could be linked back to the team level in future studies.

### Fidelity assessment challenge 4: the missing data problem

Completed goal setting worksheets and observations of goal progress presentations were used to assess fidelity receipt and enactment at workshops 1 and 2, respectively. However, at times we faced significant missing data problems. Twelve percent (11/91) of the unit teams present at the first workshop did not complete or submit their goal setting worksheets—making it impossible to use these documents to assess fidelity receipt (i.e. were they able to complete all sections of the goal setting work sheet and how well did they do it). Similarly, 46% of the unit teams (37/82) present at the second workshop did not have their assigned PowerPoint presentation prepared and, instead, these teams reported on their progress less formally, using the headings in the presentation template we provided. Because fidelity enactment of ‘intervention activities carried out by teams in their facilities’ was assessed through observation of workshop two presentations *and* examination of Powerpoint slides, assessment of this fidelity enactment component was more limited for care teams who did not use PowerPoint slides compared to teams who had prepared a PowerPoint presentation. For the post-intervention focus groups, 68% of teams (62/91) had representation in a focus group, but only 30% of individuals (33/109) who attended an INFORM workshop participated in a focus group (recall that many INFORM participants were unit managers representing multiple care units). These participation rates raised concerns regarding representativeness of the focus group data.

The described levels of missing data occurred despite various follow-up requests made to nursing home teams to reduce missing data and increase focus group participation. For instance, teams that could not attend the second workshop were asked to provide their PowerPoint slides. Similarly, to increase focus group participation rates, focus groups were conducted via teleconference, several time slots were offered to participants, and individual interviews were offered to anyone who preferred a one-on-one conversation.

Non-attendance of entire team(s) at one or more workshops (a failure to enact this intervention component) also lead to missing fidelity data related to other intervention components—something that needs to be accounted for in fidelity assessment.

### Fidelity assessment challenge 5: responding to, and treating, fidelity ‘failures’ and ‘deviations’ and lack of an overall fidelity assessment scheme

As noted above, teams did not always attend all three workshops, which represents a failure to enact this component of the intervention. There were times when only one team showed up to what were supposed to be multi-team workshops. This meant the social interaction/peer support component of the intervention was not ‘delivered’, constituting a fidelity delivery ‘failure’ (and, of course, components that are not delivered, cannot be received by participants either). Similarly, webinar technology failed to work properly in some instances where teams had poor technology infrastructure in their facility. This was recognized, in real-time, as a threat to fidelity delivery and teams participated via teleconference instead. However, this change led to reduced interaction among peer teams and constitutes a fidelity delivery deviation (adaptation)—and it may have affected participants’ receipt as well.

The literature offered little in the way of concrete guidance regarding how to treat these types of fidelity ‘failures’ and ‘deviations’ (adaptations) in our fidelity analysis. We resolved this challenge by adhering to the principle of fidelity of treatment design (i.e. ensuring an intervention is defined and operationalized consistent with its underlying theory) [3], and we returned to the core intervention components when assigning fidelity ratings. As such, a team that ended up alone at a workshop was assigned a value of ‘0’ for the fidelity item reflecting whether inter-team activities were delivered. Teams at a workshop with at least one other team were assigned a value of ‘1’, ignoring whether teams joined via web or telephone technology. This decision was guided by adherence to the core components of the intervention, but it was also a pragmatic decision in that there would be insufficient variation if this variable was coded as anything other than binary.

A related challenge was the lack of guidance regarding how to bring fidelity data together into an overall fidelity assessment scheme. We considered whether variables reflecting delivery (or receipt, or enactment) of each of the core components of the intervention (e.g. delivery of inter-team activities, delivery of goal-setting strategies) can or should be combined into an overall ‘fidelity delivery’ score. Ultimately, because items will reflect delivery of different core components of the intervention, we determined it is not appropriate to scale delivery items together and combine them into a single fidelity delivery construct. We therefore used separate variables to reflect ‘delivery’ of each of the core intervention components. The same rationale was applied to the question of whether to combine delivery, receipt, and enactment variables into an overall fidelity score. Because these items reflect different aspects of fidelity rather than a single fidelity construct, they were not scaled together.

### Fidelity assessment challenge 6: ensuring that fidelity assessment does not threaten internal validity

Focus groups designed to elicit information regarding fidelity receipt and enactment were initially planned for midway through the intervention. These were cancelled due to concerns that the focus group discussion would become part of (i.e. strengthen) the intervention. Instead, we capitalized on existing elements of the intervention in order to assess fidelity receipt and enactment in real time during the intervention. As noted above, goal-setting worksheets from workshop 1 were coded and used as measures of fidelity receipt. Structured observations of team progress presentations (during workshop 2) captured data on a team’s comprehension and implementation of INFORM activities designed to increase care aide involvement in formal team communications about resident care. These data were, in turn, utilized as measures of fidelity receipt and enactment, respectively.

## Recommendations

The fidelity assessment challenges we encountered in the INFORM trial, and the resolutions we employed, suggest a set of guidelines that can be broadly applicable to fidelity assessment in group-level process change interventions. The proposed guidelines can help overcome (a) conceptual, (b) methodological, and (c) practical issues that continue to pose challenges for rigorous fidelity assessment in pragmatic trials.

### Recommendations for improving *conceptual* approaches to fidelity assessment

A recent paper that detailed the development and testing of a measure of Fidelity of Implementation (FOI) in four settings concluded that “FOI is an important but complex phenomenon that can be difficult to measure.” [10] Indeed, Century and colleagues note that “evaluators, have yet to develop a shared conceptual understanding of what FOI is and how to measure it...” [9]. Bellg’s delineation of fidelity delivery, receipt, and enactment is comprehensive and valuable for guiding fidelity assessment. Its value over other fidelity frameworks lies, at least in part, in its inclusion of enactment as a component of fidelity, rather than a moderator of it [7]. The enactment category is particularly important as it draws attention to and reflects crucial implementation challenges teams face as they participate in complex interventions. Notwithstanding very recent fidelity work by Walton and colleagues [5, 40] which attends well to fidelity receipt and enactment (collectively referred to as intervention engagement), recent reviews found that few studies use a fidelity framework and few studies have measured fidelity delivery, receipt, *and* enactment [2, 14]. This first guideline is directed towards strengthening conceptual models of fidelity through empirical work on measurement of fidelity and examination of its relationship to effectiveness.

*Guideline C1: Little theoretical or empirical guidance is available to inform operationalization of fidelity delivery/receipt/enactment or to inform their combination and link to effectiveness results; researchers need to be mindful of this and are encouraged to undertake empirical research or theoretical work to help close the gap*. Although studies, including ours, recognize and attempt to assess the three discrete elements of fidelity (e.g. delivery, receipt and enactment), we experienced that it is not always clear how to categorize even seemingly straightforward intervention components, such as attendance at a workshop which could reasonably be considered fidelity delivery or fidelity enactment. Therefore, operationally, it may not always be possible to obtain distinct fidelity scores for delivery, receipt, and enactment. This, however, raises a conundrum—while it is sometimes difficult to clearly separate three types of fidelity, it is important to do so to examine whether different aspects of fidelity delivery, receipt, or enactment may be more or less predictive of intervention success.

Similarly, complex interventions have multiple potentially active components and it is important to assess fidelity of each of the core components of an intervention. Component-based measures of fidelity can be entered as covariates in models examining intervention effectiveness to provide valuable information on the particular mechanisms within complex interventions that have the most impact on effectiveness. Other analysis to understand which components of an intervention are robust to fidelity deviations (i.e. still causes a desired effect of reasonable size even when fidelity deviations are present) and which are not can provide important knowledge for intervention replication. Together, the above analyses can shed light on how to best integrate fidelity data with trial outcomes analysis thereby helping to provide important knowledge about ‘how’ and ‘why’ an intervention works or fails to work [6].

On the other hand, given the practical challenges of conducting detailed assessments of fidelity within interventions discussed throughout this paper, it is also important to explore combining measures of fidelity delivery, receipt and enactment, or measures of fidelity to core intervention components, into a single or smaller number of more global fidelity measures. Global fidelity measures can help researchers draw unequivocal conclusions about the effectiveness of an intervention in group-level or other interventions that do not have large samples and therefore do not have enough degrees of freedom to incorporate large numbers of fidelity variables in trial effectiveness analysis. Researchers and intervention developers will need to carefully weigh these trade-offs between global and more specific measures of fidelity as they plan and carry out fidelity assessments.

### Recommendations for improving fidelity assessment *methodology*

Our results suggest three guidelines for improving methodological rigour in fidelity assessment. *Guideline M1: Use multiple methods of fidelity assessment and build redundancy into assessment of aspects of fidelity that are under participants’ control (receipt and enactment)*. Few studies use multiple methods for measuring each aspect of fidelity [2] (see Toomey et al. [41], for a recent exception). Use of methods triangulation [42] is encouraged when assessing fidelity receipt and enactment in order to reduce the biases associated with any one approach to fidelity assessment [4]. In their study of fidelity of a nurse practitioner case management program designed to improve outcomes for congestive heart failure patients, Keith et al. found inconsistencies in different provider groups’ perceptions of whether certain program components were implemented [10]. Triangulation is a post-positivist tool that recognizes different stakeholders will have different perspectives on a shared phenomenon. We used observation, document analysis, and post-intervention focus groups to assess fidelity receipt and enactment. This kind of methods triangulation strengthens credibility of results [5] and creates redundancy of measurement which is helpful for limiting the amount of missing fidelity data. For instance, when INFORM participants goal-setting worksheets weren’t returned at workshop one, we were able to use progress presentations from workshop two to gauge fidelity receipt.

*Guideline M2: Devote attention to ensuring fidelity data are complete (i.e. low rates of missing data).* In their recent systematic review of fidelity measure quality in 66 studies, Walton and colleagues found only 1 study that reported on missing data. Complete fidelity data eliminates potential non-response bias in fidelity assessment. If only ¼ or ½ of participants take part in post-intervention interviews, their data may not be representative of all intervention participants (e.g. they may be the most highly engaged or those most frustrated by the intervention). The preceding guideline (M1) will help to achieve more complete fidelity data. When fidelity assessment data cannot be captured for all study participants, obtaining data on reasons for non-response is another helpful mitigation strategy. We obtained data on reasons for non-participation in the INFORM post-intervention focus groups that revealed non-participation was mostly due to scheduling rather than one’s opinions about, or enactment experiences with, the intervention. These data can provide confidence in the representativeness of the focus group data. Using fidelity assessment approaches, such as checklists or observation, that do not rely on participant responses can also improve fidelity data completeness.

*Guideline M3: Measurement*—*Particular attention needs to be paid to psychometric properties of fidelity measures and unit of analysis issues in group-level interventions*. Because fidelity measures tend to be intervention-specific, validated measures of fidelity are not found in the literature and measurement properties such as reliability or even the dimensionality of fidelity are unlikely to be broadly established. Efforts to examine reliability (e.g. using interrater reliability) and to justify the dimensions of fidelity that are used in a study are necessary for strengthening the quality of fidelity studies. Inter-rater agreement regarding fidelity has been carefully examined and high levels demonstrated for *less* complex interventions such as exercise programs designed to prevent lower limb injuries in team sports [43]. However, low Kappas we reported above when assessing agreement in the coding of our initial set of goal setting worksheets (to arrive at a measure of fidelity receipt) are consistent with initial levels of inter-rater agreement regarding fidelity in other complex interventions such as the Promoting Independence in Dementia (PRIDE) study [5] and the Community Occupational Therapy in Dementia-UK intervention (COTiD-UK) [40]. In more complex interventions, the work required to establish inter-rater reliability is often onerous as it may require coding of lengthy transcripts or lengthy observations and arriving at agreement. So, even when primary study measures are validated, intervention study teams are reminded of the need to (1) earmark researcher time for the fidelity assessment process and (2) include researchers with expertise in measurement to help guide any quantitative approaches to fidelity measurement.

Another important psychometric consideration is that fidelity is assessed at the same unit of analysis as the intervention being studied (i.e. at the group level in group interventions) so that fidelity data can be integrated with trial effectiveness analysis [9, 14]. However, measurement level also needs to be appropriate for any construct being measured [44] which means that in group-level interventions, individual-level assessments may sometimes be necessary to obtain a fidelity score at the group-level. For instance, satisfaction can be considered part of fidelity [10] and is typically an individual-level construct. Intervention satisfaction should therefore be measured at the individual level and then aggregated to the group level and reported not as “*team* satisfaction” but as “the proportion of *individuals* satisfied with the intervention”.

Where obtaining fidelity data at the individual or group level is not feasible, fidelity should be assessed for each study arm and in control conditions—something that is not typically done but helps to ensure treatment is absent in the control group [4]. In INFORM, we conducted interviews with a small number of participants from the standard feedback arm to assess possible threats to internal validity such as history and contamination that might have impacted our primary study outcome for all study arms.

Overall, methodological rigour associated with sampling, measurement, analysis, missing data, etc., needs to be attended to and reported for fidelity assessments just as we do in research more broadly. Failure to provide this level of rigour in fidelity assessment reflects the curious double standard noted by Peterson [45] where we carefully assess treatment outcomes (dependent variables), but rarely assess implementation (independent variables). While the need for rigour and psychometric assessment of fidelity measures continues to be highlighted, detailed examples of careful attention to psychometric qualities of fidelity scales are more and more evident in the literature. For an example, see the recent special issue on fidelity scales for evidence-based interventions in mental health [46]. The detailed approach used to develop quality measures of fidelity in the PRIDE study [5] provides another valuable example.

### Recommendations for improving *practicality*

It is evident from the guidelines suggested above that rigorous assessment of intervention fidelity can quickly become an additional, resource intensive ‘study within a study’. In their systematic review of *actual* measures used to monitor fidelity delivery, receipt, and enactment in complex interventions, Walton and colleagues [2] identified the high level of resources (time demands on study participants and those assessing fidelity as well as cost) devoted to fidelity assessment. In addition, only 25% of studies they reviewed reported on practical issues in fidelity measurement. Others have also noted that few studies report on *practical* aspects of fidelity measurement [15] and a recent study noted that lack of practical guidance was cited as a barrier to addressing/reporting intervention fidelity in trials by more than two thirds of researchers surveyed [47]. The last two guidelines are directed toward strengthening the *practicality* of Fidelity Assessment.

*Guideline P1: In multi-site interventions delivered by a single, trained resource person, assessment of fidelity receipt and enactment requires more attention and resources than assessment of fidelity delivery*. This guideline is critical as it diverges from conventional fidelity assessment practice which emphasizes delivery. While multi-site interventions require high fidelity in intervention delivery, using a single program deliverer (or team of deliverers) across intervention delivery sessions, and training them, is likely to ensure consistency of delivery. In these instances, applying observations or video/audio recording and coding (the gold standard for assessing fidelity delivery [3]) would be impractical as it would be unnecessarily resource intensive. Less onerous assessment methods such as simple fidelity checklists are sufficient in these instances [16] and can be analysed in a timely manner (e.g. immediately after the intervention delivery sessions), permitting rapid reaction to delivery deviations to minimize such deviations in subsequent sessions. Where an intervention cannot be delivered by the same person(s) in all sites, the need to measure fidelity delivery using more onerous methods of observation or recording will depend on the following considerations: difficulty of intervention delivery, training opportunities, likelihood of delivery deviations, and what leeway program deliverers can have to vary/adapt delivery before a deviation must be considered a fidelity failure. Ultimately, the relative emphasis on assessment of fidelity delivery, versus receipt and enactment, requires careful consideration and will depend on the nature of the intervention.

*Guideline P2: Build opportunities for assessment of fidelity receipt and enactment directly into the intervention.* Given the potential for fidelity assessment to become quite onerous, this guideline promotes creating opportunities for participants to ‘demonstrate’ their receipt and enactment of intervention skills and permits these aspects of fidelity to be naturally observed, yielding fidelity data that are less prone to social desirability bias than directly asking intervention participants about receipt or enactment [3]. Natural observation of participants demonstrating intervention behaviours is important [4] but was not found in any studies included in a recent review of measures used to monitor fidelity in complex health behaviour interventions [2].

In INFORM, we capitalized on opportunities to naturally observe fidelity receipt and enactment for each team when we collected and analysed their goal-setting worksheets from workshop one and when we observed goal progress presentations during workshop two. Collecting fidelity data for every team during a few intensive observation periods in each workshop was efficient and practical, particularly for a multi-site intervention where extended periods of observation in dispersed locations are costly and often infeasible. We suggest that building these kinds of opportunities for fidelity assessment *directly into the design of the intervention* may be the most valuable way to improve practicality of fidelity measurement.

In addition, participants’ ‘demonstration’ of intervention skills (i.e. by completing a worksheet or delivering a presentation on progress) provides what Kirkpatrick [48] calls level 2 outcomes of training interventions. Level 2 outcomes reflect the degree to which learners acquire knowledge and skills by allowing participants to demonstrate competence (knows how to do something) and performance (shows how) [49]. Level 2 outcomes are seen as more valuable than level 1 outcomes which reflect participants’ satisfaction with a program but do not reflect whether they can apply what they learned.

Table 3 summarizes the six guidelines proposed above and suggests which of the six fidelity assessment challenges each guideline can help with.

**Table 3:**
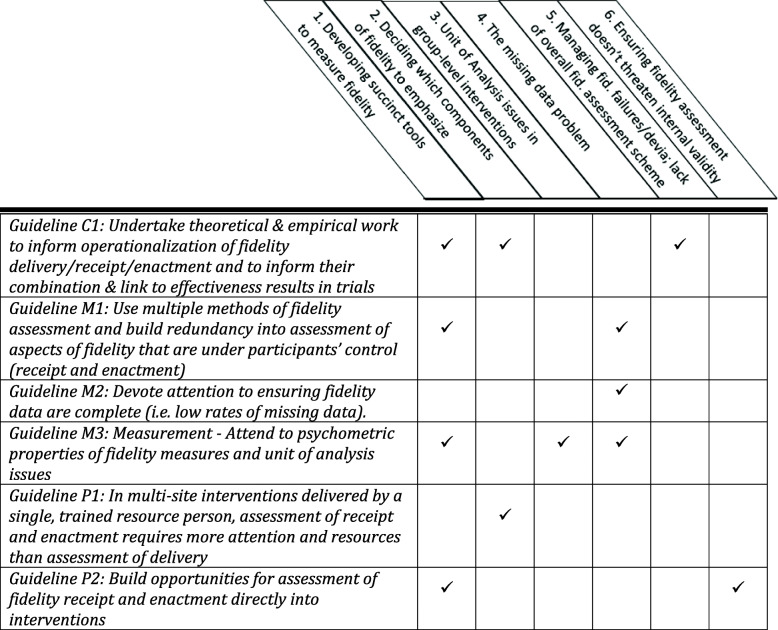
Application of fidelity assessment guidelines to identified challenges

## Conclusions and future research

There is a growing recognition of the importance of fidelity assessment in trials and the need for greater investment to fill knowledge gaps in this area. This paper seeks to contribute knowledge regarding some key conceptual, methodological, and practical fidelity challenges. However, additional research is required to deepen understanding of fidelity assessment in complex, group-level interventions and several unanswered questions remain that would benefit from additional research. First, to what extent is fidelity an *additive model* comprised of various fidelity components and can we provide empirical support for Bellg’s fidelity framework that distinguishes delivery, receipt, and enactment? Our experience suggests even the concepts of delivery, receipt, and enactment are complex and multi-faceted. For instance, enactment of intervention behaviours often requires actions throughout an intervention—in the case of INFORM enactment requires front-line managers to complete preworkshop material, attend workshops, set goals, and develop and implement strategies for increasing care aide involvement in formal care communications. Enactment may also involve multiple actors—in INFORM, high enactment would include having care staff attend formal care communication meetings when invited and having other staff groups behave in a manner that is accepting and supportive of their participation. What about the relationship between delivery, receipt, and enactment? Delivery and enactment seem to be behavioural and can be measured as actions whereas receipt is a cognitive process. And while delivery and enactment behaviours could theoretically occur on their own, receipt cannot take place without delivery.

Second, can fidelity be assessed along a continuum from low fidelity to high, and how would such a continuum be operationalized? Similarly, what constitutes ‘low’ and ‘high fidelity’—Mowbray [4] points out that the meaning of low fidelity varies—is it usual care, absence of an intervention component, or incorrect use of that component? Finally, how can we use the concepts of ‘drift’ and ‘core’ and ‘peripheral’ components of an intervention to help us understand and measure fidelity *failures* and *adaptations*—areas that become particularly important when we think about scaling interventions up for larger evaluations. Careful delineation of the core components of an intervention (those clearly rooted in theory that are essential, and integral to understanding the mechanisms of impact in an intervention) is one critical step in outlining the boundaries of what constitutes fidelity and acceptable adaptations. Such a delineation can help with understanding about how to maintain fidelity (and carry out fidelity measurement) once interventions are scaled within complex systems impacted by numerous contextual influences.

This paper contributes knowledge regarding important conceptual, methodological, and practical considerations in fidelity assessment and suggests concrete guidelines to help researchers examine fidelity in complex, group-level interventions. Greater attention to fidelity assessment and publication of fidelity results through detailed case reports such as this one are critical for improving the quality of fidelity studies and, ultimately, the utility of published trials. Ongoing fidelity research can assist with operationalization of fidelity in complex trials and can increase understanding of how and why complex behavioural interventions work or fail to work.

## Supplementary Information

**Additional file 1.** Fidelity data collection tools

## Data Availability

The data used for this article are housed in the secure and confidential Health Research Data Repository (HRDR) in the Faculty of Nursing at the University of Alberta (https://www.ualberta.ca/nursing/research/supports-and-services/hrdr), in accordance with the health privacy legislation of participating TREC jurisdictions. These health privacy legislations and the ethics approvals covering TREC data do not allow public sharing or removal of completely disaggregated data from the HRDR, even if de-identified. The data were provided under specific data sharing agreements only for approved use by TREC within the HRDR. Where necessary, access to the HRDR to review the original source data may be granted to those who meet pre-specified criteria for confidential access, available at request from the TREC data unit manager (https://trecresearch.ca/about/people), with the consent of the original data providers and the required privacy and ethical review bodies. Statistical and anonymous aggregate data, the full dataset creation plan, and underlying analytic code associated with this paper are available from the authors upon request, understanding that the programs may rely on coding templates or macros that are unique to TREC.

## Abbreviations

INFORM: Improving Nursing Home Care through Feedback On perfoRMance data
TREC: Translating Research in Elder Care
