# Supplementary material for: Fidelity is not easy! Challenges and guidelines for assessing fidelity in complex interventions
Source: Trials. 2021 May 29;22:372. doi: 10.1186/s13063-021-05322-5 (PMC8164256; doi:10.1186/s13063-021-05322-5)
Supplement: Supplementary file 1 — Additional file 1. Fidelity data collection tools [file 13063_2021_5322_MOESM1_ESM.pdf]

**Completed by:**

**Date:** **Time:** **Region:**

**INFORM Intervention Delivery Checklist**

- ❑ Facilitator, researcher and decision maker arrived as planned
- ❑ All agenda items were addressed in the intended order, using the defined methods and addressing the defined topics
- ❑ All group-work sessions took place and received the allotted time
- ❑ Timing of the workshops was as intended (no delayed start, overrun, early ending)
- ❑ Blinding was maintained (there was no discussion of the study arms)
- ❑ Instructions for reporting back at the support workshop were provided to participants

**Workshop Section Specific Fidelity**

- ❑ Introduction to TREC and INFORM completed as developed

Comments:

- ❑ Team Planning Session 1: Intro Goal Setting presented as developed and completed as instructed

Comments:

- ❑ Feedback and Measures Section completed as developed

Comments:

- [ ] Team planning Session: Developing Goals and Measures presented as developed and completed by as instructed

| Comments |
|---|
| |

- [ ] Triad Feedback Exercise presented as developed and completed by as instructed

| Comments |
|---|
| |

- [ ] Team Planning Session 3 presented as developed and completed by as instructed

| Comments |
|---|
| |

- [ ] Next Steps presented as developed

| Comments |
|---|
| |

Record any notes here to capture deviations from the workshop protocol or any differences you noticed between the workshops

| |
|---|
| |

**TO BE COMPLETED POST-WORKSHOP BASED ON EXIT SURVEYS**

___/___ Proportion of managerial teams that received the pre-workshop materials (the Workbook and the Reflection exercise)? (compiled from GSW Exit Survey, question 4)

___/___ Proportion of managerial teams who attended the full workshop (from observation)

___/___ Proportion of managerial teams that handed in their Goal Setting Worksheet after the workshop

___/___ Proportion of handed in Goal Setting Worksheets with all sections completed

___/___ Proportion of handed in Goal Setting Worksheets with identified simple measures managerial teams can use to evaluate goal progress

___/___ Proportion of managerial teams that reported they intend to make changes in their setting because of the workshop

___/___ Proportion of managerial teams who indicated that instructions they received for reporting back at the support workshop were clear (compiled from GSW Exit Survey, question 10).

**Completed by:**

**Date:** **Time:** **Region:**

| Topic to work on | Identify possible reasons for the gap in performance and on site opportunities/resources to support improvement | Initial thoughts on how we might improve performance |
|---|---|---|
| *Formal Interactions (care aid involvement in communications about resident care* | | |
| **Goal (Is the goal: Specific, Measurable, Attainable, Relevant, Time-bound?)** | **Strategies (Steps or activities required to achieve goal)** | **Measures (Concrete criteria for measuring progress toward the achievement of the goal)** |
| | | |

**INFORM Goal Setting Workshop – Evaluation Form**

Thank you for participating in this workshop. This exit survey will take 3 to 5 minutes to complete. Your feedback will help us to evaluate the workshop.

Date: ____________________ ID Sticker ____________________________________

1. Position Title:
   Please select ***one*** of the following options. Pick the one that best reflects your position.
   - ❑ Manager of care services within a facility: (e.g. director of care, facility administrator)
   - ❑ Manager of care services within a facility at the unit level (e.g., care manager, unit manager)
   - ❑ Manager of care services on health region or province level
   - ❑ Regulated care provider (e.g., registered nurse, licensed practical nurse, therapist, social worker, etc.)
   - ❑ Unregulated care provider (e.g., health care aide, personal care attendant, special care aide, nursing attendant, etc.)
   - ❑ Other (please specify): ____________________

2. How long have you worked in residential care? Years: __________ Months: __________

3. How long have you worked at your current facility? Years: __________ Months: __________

4. Please select the option that best reflects your opinion about the various workshop components:

---

**A. Pre-workshop Reflection Exercise**

A1. Did you receive the pre-workshop reflection exercise?
❑ Yes;
❑ No; Go to Question B

A.2. Did you complete the pre-workshop reflection exercise?
❑ Yes;
❑ No; Go to Question B

| | Strongly Disagree | Disagree | Neither Agree Nor Disagree | Agree | Strongly Agree |
|---|---|---|---|---|---|
| A.3. The pre-workshop reflection exercise was valuable | ❑ | ❑ | ❑ | ❑ | ❑ |

---

**B. Goal Setting Workbook**

B.1. Did you receive the Goal Setting Workbook?
❑ Yes;
❑ No; Go to Question C

B.2. Did you review the Goal Setting Workbook?
❑ Yes;
❑ No; Go to Question C

| | Strongly Disagree | Disagree | Neither Agree Nor Disagree | Agree | Strongly Agree |
|---|---|---|---|---|---|
| B.3. The Goal-setting Workbook that was sent to me before the workshop was valuable | ❑ | ❑ | ❑ | ❑ | ❑ |

---

| | Strongly Disagree | Disagree | Neither Agree Nor Disagree | Agree | Strongly Agree |
|---|---|---|---|---|---|
| C. The introductory presentation about INFORM was valuable | ❑ | ❑ | ❑ | ❑ | ❑ |
| D. The presentation on S.M.A.R.T. goals was valuable | ❑ | ❑ | ❑ | ❑ | ❑ |
| E. The presentation on developing measures was valuable | ❑ | ❑ | ❑ | ❑ | ❑ |
| F. Working with my team on goal development helped with setting measureable performance goals | ❑ | ❑ | ❑ | ❑ | ❑ |
| G. Discussions and feedback from people on other teams helped with setting measureable performance goals | ❑ | ❑ | ❑ | ❑ | ❑ |
| H. Advice from the facilitator and research team members helped with setting measureable performance goals | ❑ | ❑ | ❑ | ❑ | ❑ |
| I. The content of this workshop was relevant to my day-to-day work | ❑ | ❑ | ❑ | ❑ | ❑ |
| J. I am satisfied with this workshop experience overall | ❑ | ❑ | ❑ | ❑ | ❑ |
| K. I intend to make changes in my setting because of this workshop | ❑ | ❑ | ❑ | ❑ | ❑ |

5. At your facility, how many units will you be leading the implementation of goals? ❑ 1 ❑ 2 ❑ 3 ❑ 4+

6. The workshop will help me carry out changes in my setting. ❑ Yes ❑ No

7. If yes, what goal did you set? What changes do you plan on making?

8. Did you identify a way to measure your goal? If so, please describe?

9. What were the <u>most useful</u> aspects of the Workshop? The <u>least useful</u>? What could have made this workshop more useful to you?

10. Have you received training in quality improvement techniques? ❑ Yes ❑ No
11. Have you ever led a quality improvement project? ❑ Yes ❑ No
12. The instructions for reporting back at the support workshop were clear ❑ Yes ❑ No

**THANK YOU FOR YOUR PARTICIPATION!**

Translating Research in Elder Care

## Observational Data template for Workshop Presentations

*INSTRUCTIONS: The observation template below outlines the things that should be captured by the observers for each support workshop presentation (Section 1) and for each workshop as a whole (Section 2).*

**SECTION 1:** You should also have copies of each group's slides. If you print these and use them to make comments as you listen to each presentation please be sure to ALSO provide your overall impressions on this form at the close of the support workshop. Please submit all of your observations, whether captured on this form or on a specific slide deck

Q1. There are several stages teams go through as part of implementing an action plan to try to achieve their FI (Formal team communications) improvement goals. Which of the following stages did each team complete? For each column, check all that apply:

| **INFORM Implementation Stage** | Team 1 | Team 2 | Team 3 | Team 4 |
|---|---|---|---|---|
| | Add ID(s) | Add ID(s) | Add ID(s) | Add ID(s) |
| **Agreed** to work towards improving FI | ❐ | ❐ | ❐ | ❐ |
| **Planned** out approach or changes/improvements to be implemented | ❐ | ❐ | ❐ | ❐ |
| **Carried out** some of the planned changes on the unit | ❐ | ❐ | ❐ | ❐ |
| **Measured** the impact of changes that were made | ❐ | ❐ | ❐ | ❐ |
| **Routinized** (institutionalized) changes on the unit | ❐ | ❐ | ❐ | ❐ |

Q2. *Fidelity Enactment* refers to teams actual implementation of INFORM activities (i.e., activities to improve Formal interactions on their unit). **For each team that presented at the support workshop please provide your opinion of the extent of fidelity enactment**. (There may be just one person of a unit [or even a facility] attending/reporting back, but they are always representing a team.) *Check only one box in each row.*

| | **Very low fidelity enactment** (no/almost no activities undertaken to improve FI) | | **Moderate fidelity enactment** (reasonable efforts made to improve FI but more could have been done) | | **Very high fidelity enactment** (extensive activities undertaken to improve FI) |
|---|---|---|---|---|---|
| Team 1 | ❐$_1$ | ❐$_2$ | ❐$_3$ | ❐$_4$ | ❐$_5$ |
| Team 2 | ❐$_1$ | ❐$_2$ | ❐$_3$ | ❐$_4$ | ❐$_5$ |
| Team 3 | ❐$_1$ | ❐$_2$ | ❐$_3$ | ❐$_4$ | ❐$_5$ |
| Team 4 | ❐$_1$ | ❐$_2$ | ❐$_3$ | ❐$_4$ | ❐$_5$ |

## SECTION 2: Overall Impressions

Provide your overall impressions of the **SMART goals** teams identified

Provide your overall impressions of teams' **strategies** for improving FI

Provide your overall impressions of the **measures** teams used to assess goal progress

Major **challenges / barriers** teams encountered as they worked towards their goals

**(pay attention to challenges / barriers mentioned by teams in general, but specifically document if the teams do or do not mention the role of regional leadership or facility leadership in this context)**

**Facilitators** – factors that helped teams to implement their action plan and overcome challenges

**(pay attention to facilitators mentioned by teams in general, but specifically document if the teams do or do not mention the role of regional leadership or facility leadership in this context)**

# INFORM Focus Group 1 Guide

**Preamble**
Thank you for participating in this conversation. We are meeting with you today because you are the manager/representative of a care unit participating in the INFORM study. In INFORM we provided you with feedback of research data collected in the TREC-TMS study, have discussed this data with you in workshops, and have assisted you in preparing an action plan defining: (a) areas for improvement on your unit, (b) goals to improve quality of care on your unit, and (c) strategies to achieve those goals. We would like to learn about your experiences with the workshops and with applying your action plan since the last workshop.

Your feedback will help us to evaluate this improvement program in particular, it will help us to better understand the data we are going to collect, your unit's progress towards the desired improvement goals, what difficulties you encountered, and what helped you during this process.

[**Notes to the interviewer]**:

- There are main *numbered* questions. Try to proceed through them in the order they appear, but also give people the freedom to move beyond the boundaries of each question to some extent.
- The bullets under each main question are ***probes*** you can use if participants have not talked about these areas in their response to the main question.
- You can also can ask additional ***probing*** questions as a follow to things you hear if you think this will provide additional insights regarding our 4 main question areas.
- The key to good focus group facilitation is picking up on and probing when relevant comments are made (saying things like "tell me more about that") and steering people back on track when they get off topic.
- Remember the importance of pausing to let people think/reflect.
- Ask for stories, as examples to help us better understand what they mean/their experiences.

**Focus Group Question Guide:**

1. Briefly describe an improvement goal you planned to work towards in INFORM and share what strategies or actions you implemented to achieve this goal.
    - To what extent were you able to apply the strategies/action plan you initially laid out in the goal setting workshop?
    - Describe any major adjustments you had to make to your strategies?
    - How would you describe your success in implementing the action plan?
    - What were the most successful change strategies that you applied and which were the least successful ones?
    - If you described a goal and an action that worked really well, was there a goal and action that did not work so well that you can describe? (Or vice versa)

2. Let's talk briefly about the Support Workshops
    - Did they help you to implement your action plan and achieve your goals?
    - To what extent did the feedback and discussion at the SW help you learn from your experience and revise your action plan?

3. Let's talk more now about the extent to which you were able to achieve the improvement goals that were defined in the workshops?
    - Were you able to create or use some simple measures during the last year to see if you were making progress towards your goals? How easy or difficult was this to do?
    - Did you have to adjust or modify your goals at any point? If so, please describe.

[State]: We have probably touched on some of this already but I'd like to focus now on barriers and facilitators.

4. Let's start by talking about major challenges / barriers you encountered working towards or achieving your goals?

5. Tell me about what helped you most to (a) implement the action plan or (b) overcome challenges?
    - [For EAF Groups ONLY]: To what extent did you make use of or find the email and telephone support component of the intervention valuable?

6. Are there other things we could have done during the workshops or between them to better help you implement your action plan and to achieve the desired change?

7. If you reflect on the entire INFORM Intervention, would you say that participation was valuable for you? Why or Why not?
    - …Did participation enable you to develop new skills?
    - …Did participation enable you to make important changes on your unit?
    - …What were the key learnings for you, resulting from participation in INFORM?
    - …What were the surprises or unexpected take-aways from this experience?

| |
|---|
| **Completed by:** |
| **Date:** **Time:** **Region:** |

# INFORM Workshop 3

## Overall implementation fidelity

*Fidelity Enactment* refers to teams' actual implementation of INFORM activities (i.e., activities to improve Formal interactions (FI) on their unit). **For each team that presented at the support workshop please provide your opinion of the extent of fidelity enactment.**

*Treat each care unit team as a separate team. If team members of various care units present together (i.e., 2+ units, only one presentation), still rate each of the teams presenting together separately. In addition to the presentations, take into account other information you have gleaned from teams throughout INFORM as you come up with your overall enactment rating for each team.*

| | **Very low fidelity enactment** (no/almost no activities undertaken to improve FI) | | **Moderate fidelity enactment** (reasonable efforts made to improve FI but more could have been done) | | **Very high fidelity enactment** (extensive activities undertaken to improve FI) | |
|---|---|---|---|---|---|---|
| | **1** | **2** | **3** | **4** | **5** | **NA** |
| **Team 1** | ○ | ○ | ○ | ○ | ○ | ○ |
| **Team 2** | ○ | ○ | ○ | ○ | ○ | ○ |
| **Team 3** | ○ | ○ | ○ | ○ | ○ | ○ |
| **Team 4** | ○ | ○ | ○ | ○ | ○ | ○ |
| **Team 5** | ○ | ○ | ○ | ○ | ○ | ○ |
| **Team 6** | ○ | ○ | ○ | ○ | ○ | ○ |
| **Team 7** | ○ | ○ | ○ | ○ | ○ | ○ |
| **Team 8** | ○ | ○ | ○ | ○ | ○ | ○ |
